# Supplementary material for: Evaluation of Cardiac Autonomic Function in Patients Undergoing Thoracoscopic Sympathetic Chain Clamping for Primary Focal Hyperhidrosis
Source: Med Sci (Basel). 2025 Aug 20;13(3):147. doi: 10.3390/medsci13030147 (PMC12372048; doi:10.3390/medsci13030147)
Supplement: Supplementary file 1 [file medsci-13-00147-s001.zip › medsci-3772037-supplementary.pdf]

FONDAZIONE
POLICLINICO UNIVERSITARIO
CAMPUS BIO-MEDICO
COMITATO ETICO

Prot. PAR 39.23 OSS

Dr. Danilo Ricciardi
Fondazione Policlinico Universitario
Campus Bio-Medico
U.O.C. Cardiology
Via Álvaro del Portillo, 200
00128 Rome

Rome, 4 June 2023

Subject: Opinion of the Ethics Committee expressed during the meeting of 31 May 2023

CLINICAL TRIAL REGISTRY NO.	2022.231
PROTOCOL CODE/ACRONYM	SWEAT AND HEART
STUDY TITLE	Study of Heart Rate Variability in Patients Eligible for Clamping of the Thoracic Sympathetic Chain for Hyperhidrosis
PROTOCOL VERSION AND DATE	Version 2.0, 22/05/2023
SPONSOR	Fondazione Policlinico Universitario Campus Bio-Medico
STUDY TYPE	Non-profit
STUDY DESIGN	Prospective, single-center observational
COORDINATING CENTER	Dr. Danilo Ricciardi – U.O.C. Cardiology, Fondazione Policlinico Universitario Campus Bio-Medico
DATE OF RECEIPT OF DOCUMENTS	22/05/2023
DATE OF MEETING	31/05/2023
ETHICS COMMITTEE OPINION PROTOCOL NO.	PAR 39.23 OSS

The Ethics Committee of the Fondazione Policlinico Universitario Campus Bio-Medico, convened in telematic session on **31 May 2023**, reviewed the documentation relating to the observational clinical study referred to above, submitted on 16/12/2022 (Clinical Trial Registry No. **2022.231**), with a request for protocol revision. The amended documentation was received on 22/05/2023.

LIST OF DOCUMENTS REVIEWED

Document Name	Version and Data
1. Sponsor's Letter of Intent	12/13/2022
2. Statement on the Observational Nature of the Study	12/13/2022
3. Statement on the Non-Profit Nature of the Study Pursuant to Ministerial Decree Nov 30, 2021	12/13/2022
4. Statement on the Units Involved in the Study	12/13/2022
5. List of Study Services_FPOL UCBM2022	12/13/2022
6. Sweat and Heart Protocol	v2.0 of 05/22/2023
7. Study Synopsis in Italian	v1.0 of 12/13/2022
8. Information Sheet and Informed Consent Form	v1.0 of 12/13/2022
9. Information and Consent to the Processing of Personal Data	v1.0 of 12/13/2022
10. Declaration of Conformity Kardia Mobile w KS_November 2020	11/16/2020
11. QUIVER - KardiaMobile 6L User Manual	n/a
12. Certification of CE compliance	18.01.2018
13. HRV Scientific User Guide	V4.0 dated 15.09.2022
14. Kubios-HRV-Scientific and Kubios HRV App Technical Data Sheet – Kubios	10.11.2022
15. Principal investigator's cv – dr. Ricciardi	10.05.2022
16. Public Conflict of Interest Declaration – Dr. Ricciardi	13.12.2022
17. Certificate of Attendance at the ICH GCP E6(R2) Course – Dr. Ricciardi	15.07.2020
18. Business Impact Analysis for Local Feasibility	18.04.2023

The following aspects were assessed, where applicable:
- Scientific validity and ethical justification of the study with regard to rationale, objectives, and measured endpoints;
- Representativeness of the study population, adequacy of inclusion and exclusion criteria;
- Justification of sample size and statistical plan;
- Protection of the rights, safety, and well-being of study participants;
- Adequate qualification of the investigator and availability of personnel, structural, and technological resources;
- Compliance with international and national ethical standards for biomedical research involving humans: Declaration of Helsinki (WMA, Fortaleza 2013); Good Clinical Practice (ICH/GCP); Oviedo Convention (04/04/1997);
- Adherence to confidentiality and privacy regulations in force (EU Regulation 2016/679; Italian Data Protection Code – Legislative Decree 196/2003 as amended by Legislative Decree 101/2018).

At the end of the discussion, the Ethics Committee unanimously expressed:

FAVOURABLE OPINION

Requirements following approval
The Ethics Committee reserves the right, as provided by its regulations, to conduct one or more monitoring visits during the course of the trial.

The Principal Investigator must notify the Ethics Committee, for monitoring purposes, of:
• the date of enrolment of the first subject and the end of the enrolment period;
• the progress of the study, through submission of an annual report;
• the date of study completion, as well as any premature suspension, including the reasons thereof;
• the final results of the study, through submission of a final report within one year after its completion.

The Ethics Committee must also be informed, within the timelines established by applicable regulations, of:
• adverse events, whether serious or unexpected, occurring during the study, and any factor that could affect subject safety or the continuation of the study;
• protocol deviations;
• protocol amendments, which may not be implemented without the prior written favourable opinion of the Ethics Committee on a specific amendment, except where necessary to eliminate immediate risks to subjects or where the amendment(s) concern only logistical or administrative aspects of the study.

It is also recalled that it is the explicit responsibility of the sponsor to make the research results publicly available in a timely manner, even if negative, in accordance with Ministry of Health Circular No. 6 of 02/09/2002 and Ministerial Decree of 12/05/2006.

The Ethics Committee of the Fondazione Policlinico Universitario Campus Bio-Medico, established and organized pursuant to Ministerial Decree of 08/02/2013, operates in accordance with Good Clinical Practice (ICH GCP) and the regulatory provisions governing the establishment and functioning of Ethics Committees and clinical trials.

ETHICS COMMITTEE MEETING 31 MAY 2023

Mandatory components	Qualifications (according to Ministerial Decree 08.02.2013)	Exterior/ Interior	P: Present A: Absent
Prof. Francesco Pallone	Clinician-Gastroenterologist (President)	Exterior	P
Dott. Angelo Insola	Clinician-Neurologist	Exterior	P
Dott. Stefano De Lillo	Territorial General Practitioner	Exterior	P
Prof. Bruno Nobili	Pediatrician	Exterior	P
Dott.ssa Domenica Ioele	Pharmacist	Exterior	P
Dott. Patrizio Rossi	Medical Examiner	Exterior	P
Prof. Maurizio Genuardi	Expert in Genetics	Exterior	A
Dott.ssa Anita Paoletto	Representative of the Volunteer Association	Exterior	P
Dott.ssa Alessandra Mecozzi	Pharmacist	Exterior	P
Prof. Massimo Ciccozzi	Biostatistician	Interior	P
Prof. Raffaele Antonelli Incalzi	Clinician-Geriatrician	Interior	P
Prof. Daniele Santini	Clinician - Oncologist	Exterior	A
Prof. Giuseppe Perrone	Clinician-Anatomopathologist	Interior	A
Dott. Lorenzo Sommella	Health Director	Interior	A
Dott. Pierantonio Menna	Pharmacologist	Interior	A
Prof. Vittoradolfo Tambone	Bioethics Expert	Interior	A
Dott. Andrea Di Mattia	Pharmacist expert in medical devices	Interior	A
Dott.ssa Anna De Benedictis	Representative of the health professions	Interior	A

The investigator or members of the Committee who have a direct or indirect conflict of interest with the study did not participate in the discussion, opinion, or vote.

Quorum: 10 out of 18

The President of the Ethics Committee
(Prof. Francesco Pallone)

FRANCESCO
PALLONE
04.06.2023
15:48:34
GMT+01:00

INFORMATION SHEET AND INFORMED CONSENT FORM FOR PARTICIPATION IN AN OBSERVATIONAL STUDY

**Official Study Title: STUDY OF HEART RATE VARIABILITY IN PATIENTS CANDIDATES FOR THORACIC SYMPATHETIC CHAIN CLAMPING FOR HYPERHIDROSIS**
**Simplified Title: Heart Rate Analysis in Patients with Hyperhidrosis Undergoing Surgery**
**Identification Code: Sweat and Heart**
**Coordinating Center and Study Coordinator (if multicenter)**<br>Coordinating Center: PUCBM
**Facility/Setting Where the Study Will Be Conducted: PUCBM**
**Principal Investigator:** Dr. Danilo Ricciardi
**Co-Investigators:**<br>• Dr. Francesco Picarelli<br>• Gaia Loiacono (student)
**Sponsor/Funding Organization**
**Ethics Committee**

This document consists of the following sections:
A. **INTRODUCTION**
B. **INFORMATION SECTION: STUDY SUMMARY - KEY INFORMATION**
C. **INFORMATION SECTION: FURTHER DETAILS**
D. **CONSENT EXPRESSION SECTION**

Dear Sir/Madam,
*The information contained in this information sheet is very detailed. We ask you to agree to participate in the study **ONLY** after carefully reading this information sheet and having a **THOROUGH DISCUSSION** with a member of the research team, who will dedicate the **NECESSARY TIME** to ensure you fully understand what is being proposed to you.*

INTRODUCTION

Dear Madam/Sir,
We invite you to participate in the clinical study titled:

Below, we explain it to you
You have the right to be informed about the purpose and characteristics of this observational research study so that you can make an informed and voluntary decision about whether to participate.
This document aims to inform you about the nature of the study, its objectives, and what your participation will involve, including your rights and responsibilities.
We invite you to read carefully the information below. The researchers involved in this project, listed at the beginning of this document, are available to answer any questions you may have. No question you think of is trivial—please do not hesitate to ask!
In addition to discussing with us, you may also talk about the proposal in this document with your family doctor, your relatives, or other trusted persons. Please take all the time you need to decide.
You can take home an unsigned copy of this document to think it over or discuss it with others before making a decision.
If you decide not to participate in the study, you will still receive the best possible care for your condition/disease.
Your refusal will in no way be interpreted as a lack of trust.

IF APPLICABLE:
To facilitate the understanding of this document, the study center provides a cultural mediator who can convey the information in the manner and language most suitable for you.

IF APPLICABLE:
If you are unable to sign the informed consent, consent can be recorded using appropriate alternative methods, such as audio or video recordings.

Once you have read this form, had any questions answered, and decided to participate in the study, you will be asked to sign a consent form, of which you will receive a paper copy.

The Principal Investigator

A. **INFORMATION SECTION**
GENERAL SUMMARY OF THE STUDY: KEY INFORMATION

This section aims to briefly present the key aspects of the clinical study we are proposing you to oin. The following sections will provide more detailed information so that you can give or withhold our fully informed consent to participate in the study.

1. **Why am I being asked to participate in this study?**
We are asking you to participate in this clinical study because we are conducting research on the autonomic nervous system in patients with primary focal hyperhidrosis and/or facial erythema, aged between 18 and 50 years.

2. **What are the study objectives?**
The overall objective of the study is to document a systemic cause of hyperhidrosis related to the autonomic nervous system rather than a peripheral cause. Specifically, this research aims to obtain data on heart rate variability to use it as a diagnostic marker.

3. **How many centers and patients will participate? What is the expected duration of the study?**
The study will last 24 months, with a total of 100 patients from this polyclinic participating.

4. **Is participation voluntary?**
You are free to decide whether or not to participate in the study. Even after agreeing, you can change your mind at any time.

5. **What happens if I decide not to give my consent to participate?**
If you decide not to participate in the study, you will still receive care at the clinical center treating you, and you will be treated with the best approved (non-experimental) therapeutic methods for your condition.

6. **What happens if I decide to participate?**
If you agree to participate in this study, you will undergo an initial visit to verify that your condition meets the inclusion criteria. During this visit, you will have a cardiological examination and an electrocardiogram. Throughout the study, your doctor will record specific information in an electronic Data Collection Form developed for this study, which will include data already present in your medical records (e.g., medical history) as well as any new information collected during the observation period, including any specialist reports you undergo. Participation in the clinical study will not involve any additional costs for you.

7. **What will be my commitment and responsibilities if I decide to participate?**
You must carefully follow the instructions and requests from the healthcare staff overseeing the study and ensure you attend all scheduled appointments.
(For example: Inform the study doctor about all medications you are taking, including non-conventional medicines, any side effects that occur during the study, and any visits or hospitalizations at facilities other than the study center.)

- ***Expected Benefits***
  There is no direct therapeutic benefit for you from participating in this study; however, the information collected may contribute to a better understanding of your condition and could help improve the diagnosis and treatment of people who, like you, suffer from primary focal hyperhidrosis.

- ***Potential Risks***
  Participation in the study will not expose you to any additional risks beyond those related to the usual care procedures you undergo for the diagnosis/treatment of your condition.

- ***Insurance Coverage***
  The study involves only observation and data analysis; therefore, participants will be covered by insurance under the risk management program of standard clinical practice (Civil Liability Policy with Cattolica Assicurazioni, no. 00218532300647), in accordance with current regulations.

- ***Compensation for Participation***
  No compensation or reimbursement for expenses is provided for participating in this study.

- ***Is Consent Final? Can I Withdraw?***
  Your participation in this research program is entirely voluntary, and you may withdraw from the study at any time without providing a reason and without any negative consequences on the quality of healthcare you receive.
  If you decide to withdraw, please inform one of the study doctors as soon as possible.

- ***Are there reasons why the study might be stopped without my consent (early termination)?***
  If the study is interrupted by the competent authorities or the sponsor, you will be promptly informed.

INFORMATION SECTION. FURTHER DETAILS

1. **How will my health data, including identifying information, be handled and who will have access to it during the study?**
   Your data, particularly health-related data and only to the extent necessary for the study's objectives and pharmacovigilance purposes, will be processed in compliance with European and Italian regulations on the protection of individuals regarding the processing of personal data (EU Regulation 2016/679, known as GDPR (General Data Protection Regulation), and Legislative Decree no. 196/2003 (Privacy Code) as amended by Legislative Decree no. 101/2018). All informations about the processing of your personal data is contained in the attached privacy notice
2. **How can I access the results of the study?**
   Once the study is completed and all data collected, the data will be analyzed to draw conclusions. The investigators commit to making the results available to the scientific community.
   Participants have the right to access the study results. Therefore, you may ask the study doctor to provide you with the general results of the study or inform you where and how to access them.
3. **Has the study been approved by an Ethics Committee?**
   The study protocol proposed to you has been reviewed and approved by the Ethics Committee of the Campus Bio-Medico. The Ethics Committee has verified that the study complies with the European Union Good Clinical Practice standards and the ethical principles expressed in the Declaration of Helsinki, ensuring that your safety, rights, and well-being are protected.
4. **Who can I contact for more information about the clinical study I am invited to participate in?**
   For further information and communication during the study, you can contact Dr. Danilo Ricciardi. Please report any problems that may arise during your participation in this study.
   You can reach the healthcare professional at the following contact: Cardiology Secretariat, phone: +39 06 225411612.
5. **If I participate in the study, who can I contact in case of need?**
   For any questions or unforeseen events during the study, you can contact Dr. Danilo Ricciardi.

If you wish to report any events or issues related to the study to parties not directly involved in the research, you may contact the Ethics Committee that approved the study (Policlinico Campus Bio-Medico) or the Medical Direction of the Study Center (Policlinico Campus Bio-Medico).

CONSENT TO PARTICIPATE IN AN OBSERVATIONAL STUDY

Clinical Study Title:
Acronym: Sweat and Heart
Principal Investigator: Dr. Danilo Ricciardi

I____born on__/__/__

resident in (city)_____________at (street/square)_____________________Tel.___________

domiciled at (if different from residence) ____

**1**	I declare that the nature, purpose, procedures, expected benefits, possible risks and inconveniences, as well as the alternative treatment options to the proposed clinical trial, have been clearly explained to me.	☐
**2**	I confirm that I have read and understood the information provided in this information sheet, that I have had the opportunity to ask questions to the study investigator and received satisfactory answers, and that I have had sufficient time to consider.	☐
**3**	I understand that my participation is voluntary and that I am free to withdraw at any time without giving any reason, and that if I do so, it will not affect my future care and attention from the doctor.	☐
**4**	I am aware that my clinical data may be accessed by personnel authorized by the sponsor and its delegates, as well as by regulatory authorities.	☐
**5**	I have been informed that the results will be made available to the scientific community, protecting my identity in accordance with current privacy regulations.	☐
**6**	I confirm my willingness to participate in the proposed study.	☐
**7**	I understand that, for the best protection of my health, I am aware of the importance (and my responsibility) to inform my general practitioner about the clinical trial I agree to participate in. I am also aware of the importance of providing the investigator with all relevant information about myself (medications, side effects, etc.).	☐
**8**	I confirm that I have received a copy of the information leaflet and of this informed consent form duly signed.	☐

The participant MUST personally sign and date this form.

______________________________ ____________________ ___________________________
Participant's full name Date Signature

STATEMENT OF THE PHYSICIAN OBTAINING CONSENT:

I have explained in detail the nature and purpose of the study described above and the risks involved in its conduct. I have answered all questions to the best of my ability.

____________________	____________________	____________________
**Investigator's full name**	Date	Signature

------------------- *Use the following only if applicable* ------------------

If the patient is unable to read or sign, an independent witness, who is not involved with the investigator or sponsor, must be present throughout the entire informed consent discussion. The witness must personally sign and date the informed consent statement after the form and any other written information have been read and explained to the subject, and the subject has given verbal consent to participate in the study.

I: ____

witness that Dr. ____

has thoroughly explained to Mr. ____

the characteristics of the experimental study in question, as described in the attached information sheet, and that he, having had the opportunity to ask all the questions he deemed necessary, has freely agreed to participate in the study.

______________	______________________	__________________________
**Place and Date**	**Full name**	**Witness' signature**

In cases where the subject is incapacitated, informed consent must be obtained from the legally authorized representative. Such consent should represent the presumed will of the subject and may be withdrawn at any time without any disadvantage to the subject. The subject must receive information about the trial, its risks, and benefits according to their capacity to understand.

In cases of temporary incapacity, to continue with the trial it is necessary to obtain informed consent from the patient after the restoration of their capacity to understand and make decisions.

______________	______________________	__________________________
**Place and Date**	**Full name**	**Signature of the legal representative**
